# Comparison of activity, structure, and dynamics of SF-1 and LRH-1 complexed with small molecule modulators

**DOI:** 10.1016/j.jbc.2023.104921

**Published:** 2023-06-14

**Authors:** Michael L. Cato, Emma H. D’Agostino, Racheal M. Spurlin, Autumn R. Flynn, Jeffery L. Cornelison, Alyssa M. Johnson, Rei A. Fujita, Sarah M. Abraham, Nathan T. Jui, Eric A. Ortlund

**Affiliations:** 1 Department of Biochemistry, Emory University School of Medicine, Atlanta, Georgia, USA; 2 Department of Chemistry, Emory University, Atlanta, Georgia, USA

**Keywords:** nuclear receptor, small molecule, crystallography, molecular dynamics, allostery

## Abstract

Steroidogenic factor-1 (SF-1) is a phospholipid-sensing nuclear receptor expressed in the adrenal glands, gonads, and hypothalamus which controls steroidogenesis and metabolism. There is significant therapeutic interest in SF-1 because of its oncogenic properties in adrenocortical cancer. Synthetic modulators are attractive for targeting SF-1 for clinical and laboratory purposes due to the poor pharmaceutical properties of its native phospholipid ligands. While small molecule agonists targeting SF-1 have been synthesized, no crystal structures have been reported of SF-1 in complexes with synthetic compounds. This has prevented the establishment of structure–activity relationships that would enable better characterization of ligand-mediated activation and improvement in current chemical scaffolds. Here, we compare the effects of small molecules in SF-1 and its close homolog, liver receptor homolog-1 (LRH-1), and identify several molecules that specifically activate LRH-1. We also report the first crystal structure of SF-1 in complex with a synthetic agonist that displays low nanomolar affinity and potency for SF-1. We use this structure to explore the mechanistic basis for small molecule agonism of SF-1, especially compared to LRH-1, and uncover unique signaling pathways that drive LRH-1 specificity. Molecular dynamics simulations reveal differences in protein dynamics at the pocket mouth as well as ligand-mediated allosteric communication from this region to the coactivator binding interface. Our studies, therefore, shed important insight into the allostery driving SF-1 activity and show potential for modulation of LRH-1 over SF-1.

Steroidogenic factor-1 (SF-1; NR5A1) is 1 of 48 human members of the nuclear receptor (NR) family, composed of ligand-regulated transcription factors that control the expression of genes involved in critical biological processes such as development, metabolism, and reproduction (1, 2, 3). SF-1 belongs to the NR5A subfamily, a class of monomeric, phospholipid-binding NRs (4, 5). SF-1 is critical for development of the adrenal glands, gonads, and ventromedial nucleus of the hypothalamus (VMH), where it also plays critical roles in steroidogenesis and energy homeostasis (6, 7, 8, 9, 10, 11, 12). In the adrenal glands and gonads, SF-1 controls the expression of steroidogenic genes as well as the expression of receptors for adrenocorticotropic and follicle-stimulating hormones to stimulate steroidogenesis (13, 14, 15, 16). In the VMH, SF-1 regulates energy homeostasis, although the precise genes controlled by SF-1 are unclear (17, 18). Loss of SF-1 in this tissue leads to obesity due to increased food intake, decreased energy expenditure, and impaired glucose tolerance and insulin sensitivity (19). SF-1 has also been implicated in adrenocortical carcinoma, a rare and deadly cancer (20, 21, 22, 23, 24, 25, 26, 27). Therefore, SF-1 is an attractive therapeutic target for disease states characterized by dysregulation of steroidogenesis and energy homeostasis as well as adrenocortical carcinoma.

Liver receptor homolog-1 (LRH-1; NR5A2) is the only other human NR5A and is largely expressed in tissues of endodermal origin (5). It is well characterized in the liver and intestine, where it regulates processes such as lipid and glucose homeostasis, as well as steroidogenesis (5, 28). Accordingly, this receptor has been successfully targeted to alleviate symptoms associated with diabetes (29) and inflammatory bowel disease (30) *in vivo*.

The endogenous ligands for the NR5As are believed to be phospholipids (PLs) (Fig. 1). Early crystal structures of both SF-1 and LRH-1 revealed bacterial phospholipids in the binding pocket, with the polar head engaging hydrophilic residues at the mouth of the pocket and the fatty acyl tails protruding into the largely hydrophobic pocket interior (31, 32, 33, 34). Subsequent studies identified phosphatidylcholine species which could activate both NR5As (35, 36), and dietary phospholipid 1,2-dilauroyl-sn-glycero-3-phosphocholine (DLPC) was able to activate LRH-1 *in vivo* (29). Both NR5As can also bind phosphatidylinositols with high affinity (Fig. 1) and numerous studies have found that these lipids can regulate SF-1 activity (34, 37, 38, 39, 40, 41, 42, 43). However, PLs do not make ideal therapeutic candidates as they are challenging to work with in the laboratory setting and are rapidly metabolized and remodeled *in vivo*, limiting their efficacy. There is significant interest in the development of synthetic NR5A ligands, and recent progress has produced high affinity modulators (44, 45, 46, 47). Cross-reactivity with SF-1 has been reported for some of these small molecules, and there have been several promising antagonists developed (44, 45, 48, 49, 50, 51). Nevertheless, no structure has been reported of SF-1 in complex with a synthetic modulator, preventing the establishment of a structure–activity relationship to further compound development.

**Figure 1:**
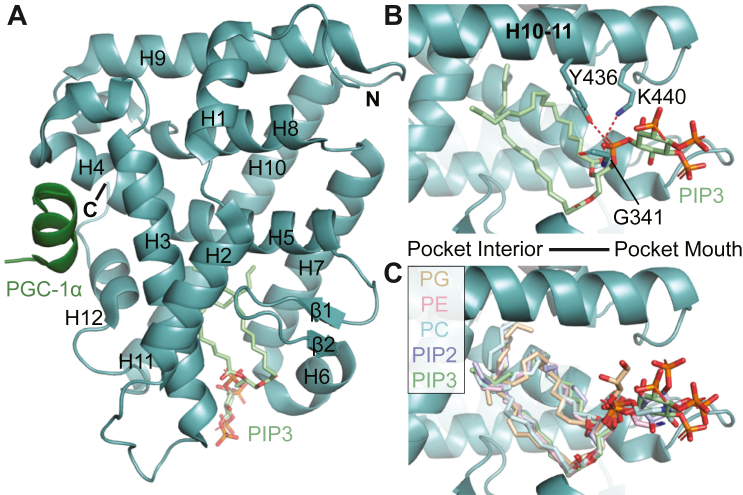
**Various phospholipid species bind SF-1 with a similar binding pose.***A*, structure (PDB entry 4QJR) of SF-1 LBD (*teal*) bound to PIP3 (*light green*). The fragment of coactivator peroxisome proliferator-activated receptor gamma coactivator 1-alpha (PGC-1α) is shown in *dark green*. *B*, PIP3 binding orientation is shown, with residue side chains (and main chain of G341) critical for contacting the phospholipid polar head group shown as *sticks*. *C*, the binding orientation of different phospholipids is shown. Each phospholipid is oriented with the polar head at the mouth of the pocket and fatty acyl tails occupying the pocket cavity. Phospholipids included are phosphatidylglycerol (“PG,” tan, PDB entry 1YOK), phosphatidylethanolamine (“PE,” *pink*, PDB entry 1ZDT), phosphatidylcholine (“PC,” *cyan*, PDB entry 3F7D), phosphatidylinositol 4,5-bisphosphate (“PIP2,” *purple*, PDB entry 4QK4), and phosphatidylinositol 3,4,5-trisphosphate (“PIP3,” *green*, PDB entry 4QJR). O = *red*, N = *blue*, P = *orange*, C = variable.

We used a library of small molecule agonists designed to target different regions of the LRH-1 binding pocket to explore SF-1 agonism as it compares to that of LRH-1. We found that deep pocket contacts are particularly useful for LRH-1 specificity, as numerous small molecules with moieties exclusively targeting this region only activated LRH-1. However, a small molecule (“6N-10CA”) that simultaneously contacts deep pocket and mouth residues demonstrated low nanomolar affinity and potency for SF-1. The stability induced by this molecule enabled us to solve the first crystal structure of SF-1 in a complex with a synthetic agonist. While the structure shows a co-occupying bacterial PL within the crystal, the electron density for 6N-10CA is unambiguous. Molecular dynamics (MD) simulations confirm that 6N-10CA makes critical contacts at the targeted polar sites but indicate that structural features at two loops situated at the pocket mouth destabilize ligand orientation in the SF-1 binding pocket. This may explain why small molecules consistently show greater affinity for LRH-1. Simulations also suggest that small molecule agonists drive greater allosteric communication from the pocket mouth to the site of coregulator binding in LRH-1. Our mechanistic studies thus reveal how SF-1 differs in its response to small molecules and offer insight into the development of selective SF-1 and LRH-1 synthetic ligands.

## Results

### NR5A synthetic agonist design

**Figure 2:**
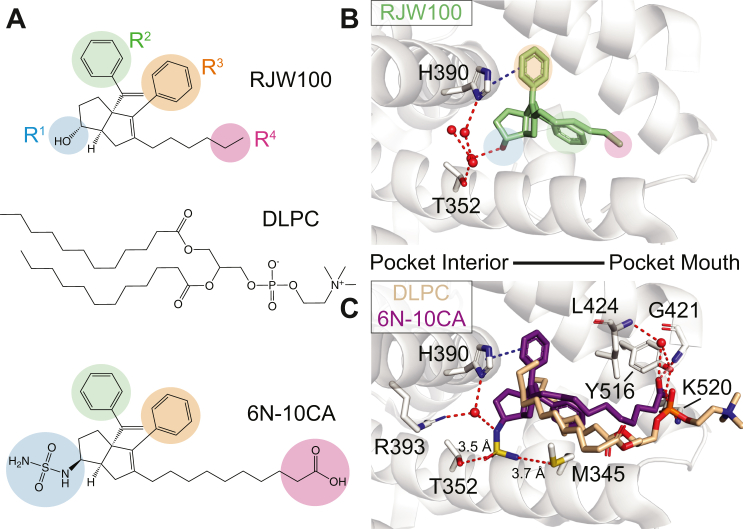
**Structure and binding pose of LRH-1 agonists.***A*, chemical structures of LRH-1 small molecule agonist RJW100 (*top*), phospholipid agonist DLPC (*middle*), and small molecule agonist 6N-10CA (*bottom*). Major substituents of synthetic ligands are highlighted. *B*, binding orientation of RJW100 (*light green*, PDB entry 5L11) is shown, with major substituents indicated as in (*A*). *C*, binding orientations of 6N-10CA (purple, PDB entry 7TT8) and DLPC (tan, PDB entry 4DOS). Interacting residue side chains (and main chains of G421 and L424) are shown as *sticks* (O = *red*, N = *blue*, S = *yellow*, P = *orange*, C = *white*). Waters are represented as red spheres, hydrogen bonds are represented as *red dotted lines*, and π–π stacking with H390 is represented as a *blue dotted line*. Only interactions with 6N-10CA are shown for simplicity.

One of the earliest NR5A synthetic agonists was RJW100, which had a bicyclic hexahydropentalene (6HP) core and activated SF-1 and LRH-1 *in vitro* (Fig. 2*A*) (45). Structural studies revealed that the R^1^ hydroxyl group indirectly engages deep pocket polar residues through a conserved water network (Fig. 2*B*) (52). With this structural information, we synthesized compounds using the RJW100/6HP scaffold that targeted polar residues deep within the pocket. Replacing the R^1^ hydroxyl with a sulfamide radically improved affinity and potency by enabling direct and indirect contacts with deep pocket polar residues (“6N;” Fig. 2) (38, 44). We next sought to increase activation by lengthening the alkyl chain (R^4^ position; Fig. 2*A*) and appending polar groups to access PL-coordinating residues at the pocket mouth (Figs. 1*B* and 2*C*) (46). This strategy was successful, resulting in improved activation and potency toward LRH-1. The best compound from this series (“10CA”) activates LRH-1 to decrease disease-associated weight loss in an *in vivo* model of ulcerative colitis (30, 46).

Recently, we combined these two design strategies into a hybrid compound (“6N-10CA”) that simultaneously accesses deep pocket and pocket mouth interactions (Fig. 2, *A* and *C*). The modifications have a synergistic effect on affinity and potency, and 6N-10CA more effectively alters LRH-1 target gene expression in HepG2 cells than its single component agonists (47).

We have also developed a library of additional RJW100 derivates and shown that some of these activate SF-1 in reporter assays (44). However, while the majority of RJW100 derivatives bind SF-1, they consistently do so with considerably lower affinity than LRH-1 (38). While our work has focused on LRH-1, the NR5A binding pockets are strikingly similar. We therefore sought to use our small molecule library to identify a useful tool for targeting SF-1, gain a better mechanistic understanding of its ligand-mediated activity, and explore what features of small molecule binding/agonism drive LRH-1 specificity.

### Biochemical characterization of small molecules

**Figure 3:**
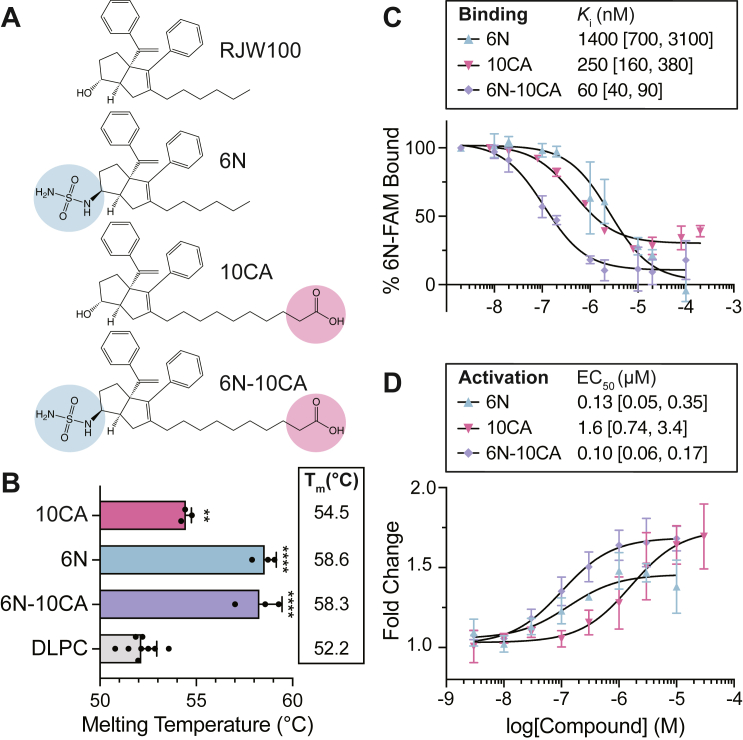
**6N-10CA displays nanomolar affinity and potency for SF-1.***A*, chemical structures of the previously published small molecules RJW100, 6N, 10CA, and 6N-10CA. The 6N sulfamide (*blue*) and 10CA carboxylic acid (*pink*) moieties are highlighted. *B*, thermal melting assay comparing effects of small molecules on SF-1 thermal stability as compared to activating PL DLPC. T_m_ = melting temperature. Data are shown as means + SD from three (small molecule) or nine (DLPC) independent experiments. Data analyzed relative to DLPC control: One-Way ANOVA with Dunnett multiple comparison’s test, ∗∗*p* < 0.01, ∗∗∗∗*p* < 0.0001. *C*, FP competition assay comparing compound binding affinity. *K*_i_ = inhibition constant. 95% confidence intervals are shown in brackets. Data shown as means ± SD from two (6N and 10CA) or three (6N-10CA) independent experiments. *D*, luciferase reporter assay showing effects of small molecules on SF-1 activity. EC_50_ = half maximal effective concentration. 95% confidence intervals are shown in brackets. Data were normalized relative to DMSO control and are shown as means ± SD from three biological replicates.

We began by comparing the functional effects of 6N-10CA with its constituent compounds, 6N and 10CA (Fig. 3*A*). Thermal shift assays revealed that all three compounds stabilize SF-1, relative to the DLPC phospholipid control (Fig. 3*B*). 6N and 6N-10CA both resulted in a positive shift in melting temperature of ∼6.5 °C, whereas 10CA shifted the melting temperature by ∼2 °C. FP competition assays (38) show that the 6N and 10CA modifications had a synergistic effect on SF-1 binding, resulting in a ∼20-fold increase in affinity over 6N and a ∼4-fold improvement over 10CA (Fig. 3*C*). The 60 nM *K*_i_ of 6N-10CA is the highest affinity reported for an SF-1 synthetic agonist, although it does not reach the picomolar affinity of 6N-10CA for LRH-1 (47). Luciferase reporter assays (Fig. 3*D*) revealed that 6N-10CA had a similar potency as 6N and was 16-fold more potent than 10CA, with an EC_50_ of 100 nM. 6N-10CA had a maximum activation of ∼1.7, which was similar to 10CA and higher than that of 6N (max = ∼1.4). Taken together, these data demonstrate that, like LRH-1, incorporation of both sulfamide and carboxylic acid moieties improves small molecule binding and activation for SF-1.

### Exploration of NR5A specificity

**Figure 4:**
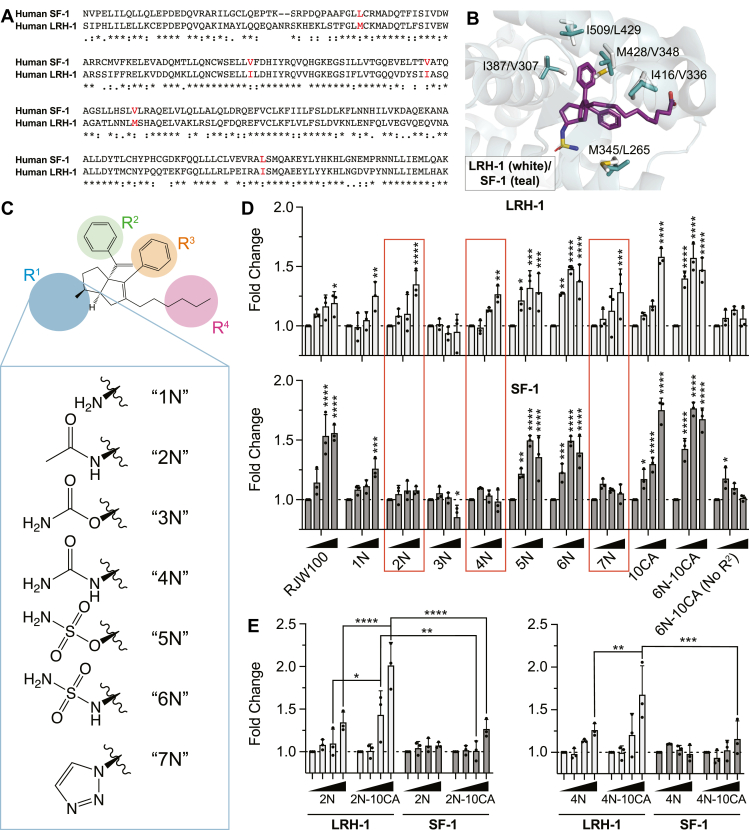
**Small molecules show specificity for LRH-1.***A*, sequence alignment of LRH-1 and SF-1 LBDs (two residues at C-termini excluded). (∗) conserved, (:) highly similar, (.) weakly similar. The LBDs of LRH-1 and SF-1 have approximately 61% sequence identity. In *red* are residues highlighted in (*B*). *B*, side chains of residues in pocket interior that differ between LRH-1 (*white*) and SF-1 (*teal*) are shown as *sticks* (O = *red*, N = *blue*, S = *yellow*, C = *white*, *teal*, or *purple*). Residues shown have side chains that face inward toward the binding pocket (residues labeled as LRH-1/SF-1). Differences at the pocket mouth are discussed later (Fig. 7). *C*, chemical structures of small molecule agonists with R^1^ modifications. *D*, LRH-1 (*top*) and SF-1 (*bottom*) luciferase reporter assays. Four bars for each ligand represent (from *left to right*) DMSO (control) and small molecule at 0.1, 1, and 10 μM. Data shown as means + SD from three biological replicates. Data normalized and analyzed relative to DMSO control: Two-Way ANOVA with Dunnett multiple comparison’s test, ∗*p* < 0.05, ∗∗*p* < 0.01, ∗∗∗*p* < 0.001, ∗∗∗∗*p* < 0.0001. Boxed in red are ligands that demonstrate specificity for LRH-1. *E*, luciferase reporter assays were used to examine LRH-1 and SF-1 activation by 2N and 4N with 10CA tail modifications. Four bars for each ligand represent (from left to right) DMSO (control) and small molecule at 0.1, 1, and 10 μM. Data shown as means + SD from three biological replicates. Data were normalized to the DMSO control and groups were compared with a Two-way ANOVA with Tukey multiple comparison’s test, ∗*p* < 0.05, ∗∗*p* < 0.01, ∗∗∗*p* < 0.001, ∗∗∗∗*p* < 0.0001.

One of the greatest challenges in NR5A small molecule development has been the ability to specifically target one receptor. The sequences between the ligand binding domains (LBDs) of LRH-1 and SF-1 are highly conserved (LBD sequence identity = ∼61%; Figure 4*A*), and the few non-identical residues in the pocket interior largely preserve hydrophobic character (Fig. 4*B*). Previous studies have noted that a few 6HP-derived small molecules are capable of specifically activating LRH-1, which suggests that specificity with this class of compounds is possible (44, 45). We used a library of small molecule agonists to identify moieties that specifically activate one NR5A over the other (Fig. 4*C*). Out of the six compounds tested, three activated LRH-1 without activating SF-1 (Fig. 4*D*). These included 2N, which was shown previously to specifically activate LRH-1 over SF-1 (44) as well as 4N and 7N. We also note that 3N reduced SF-1 activity, indicating potential utility for antagonist development. Previous studies have shown that LRH-1 is highly sensitive to modification and removal of the R^2^ substituent (44, 53). We note the same phenomenon here for SF-1, as even the highly efficacious 6N-10CA is rendered ineffective when the R^2^ group is removed (Fig. 4*D*). Therefore, deep pocket residues are an attractive region of the binding pocket for promoting specificity for LRH-1, as nearly half of small molecule variants tested drove activity of LRH-1 without activating SF-1.

Recent studies have suggested that the inclusion of the 10CA carboxylic acid tail improves small molecule potency and efficacy for LRH-1 (47). We were interested to see whether we could incorporate this moiety into small molecules that specifically activate LRH-1 without promoting cross-reactivity with SF-1. We, therefore, synthesized 2N and 4N variations (termed “2N-10CA” and “4N-10CA”) and tested them in reporter assays. Consistent with previous studies, the inclusion of the carboxylic acid tail improved agonism, and specificity for LRH-1 was largely preserved for both small molecules (Fig. 4*E*). 2N-10CA and 4N-10CA thus represent valuable tools for specifically activating LRH-1 over SF-1 and indicate that unfavorable deep pocket contacts supersede agonistic effects of SF-1 pocket mouth interactions. The only major difference deep in the pocket is the absence of a methionine (M345) present in LRH-1, which is a leucine (L265 M) in SF-1 (Fig. 4*B*). This residue has been shown to be critical for certain ligand-mediated effects in LRH-1, and mutation of this residue decreases efficacy of 2N in LRH-1 reporter assays (44, 54). We therefore hypothesized that this residue may be driving the specificity of LRH-1-specific ligands. However, 2N-10CA failed to activate the L265M SF-1 mutant, indicating that this residue is not sufficient to drive ligand-mediated activation (Fig. S1).

### Crystal structure of SF-1-6N-10CA

**Figure 5:**
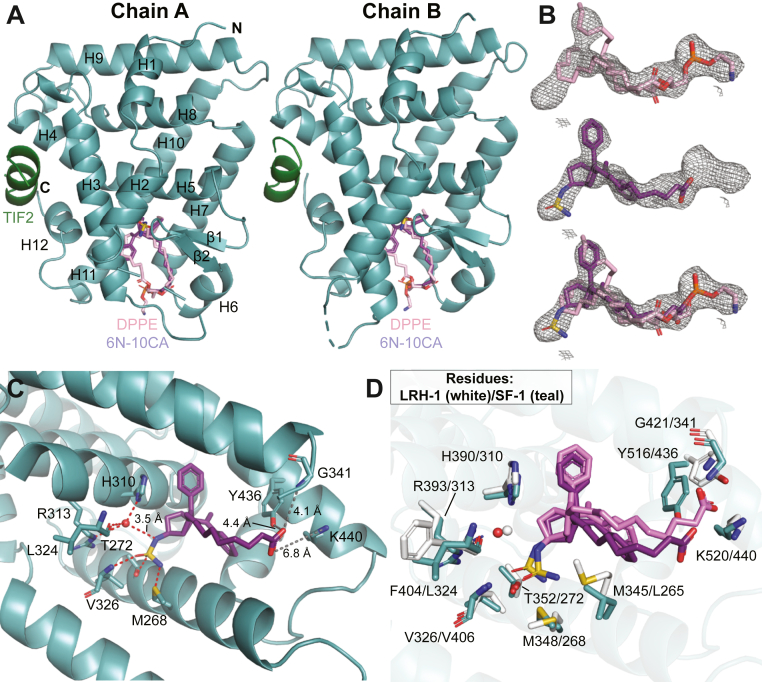
**SF-1 LBD is bound to synthetic agonist 6N-10CA and phospholipid DPPE.***A*, chains A and B of the SF-1-6N-10CA-TIF2 structure (PDB entry 8DAF). 6N-10CA and DPPE are shown co-occupying the ligand binding pocket, with the tail of DPPE overlapping with the 6N-10CA tail. Fragment of TIF2 coactivator protein is shown in green. *B*, F_o_-F_c_ omit map after deletion of all ligands in chain B of the model (contoured at σ = 2.0). *C*, binding orientation of 6N-10CA in chain B, with interacting residue side chains (and main chains of L324, V326, and G341) shown as sticks (O = *red*, N = *blue*, S = *yellow*, C = *teal or purple*). Waters are represented as *red spheres* and hydrogen bonds are represented as red dotted lines. Note that the 6N-10CA carboxylic acid tail was not within hydrogen bond distance of indicated residues. *D*, comparison of the structures of SF-1 (protein = *teal*, ligand = *purple*) and LRH-1 (PDB entry 7TT8; protein = *white*, ligand = *pink*) bound to 6N-10CA (chain B). The *red sphere* represents the mediatory water in the SF-1 structure, while the *white sphere* represents the mediatory water in the LRH-1 structure.

To visualize the 6N-10CA binding mode and understand its mechanism of action, we solved the structure of the SF-1 LBD bound to 6N-10CA and a fragment of the coactivator transcriptional intermediate factor 2 (TIF2) to a resolution of 2.59 Å (Figs. 5, S2, and Table S1). There is electron density indicating the presence of both 6N-10CA and a PL in the pocket of both monomers in the asymmetric unit, indicating partial occupancy of both ligands in the crystal (Figs. 5, *A* and *B* and S2*A*). Modeling of both ligands is supported by the strong electron density centered on the sulfamide group of 6N-10CA and phosphate moiety of the PL. We modeled in dipalmitoylphosphoethanolamine (DPPE; 16:0/16:0), which has been co-crystallized with SF-1 before (32, 33), to satisfy the PL density. Although we modeled 6N-10CA in both chains, the density for the 6HP core is more evident in chain B, and the density corresponding to the sulfamide in chains A and B becomes visible in omit maps at σ = ∼4 and σ = ∼6, respectively. Nevertheless, the relative positioning of 6N-10CA in both chains is virtually identical after refinement (Fig. S2*B*).

The 6N-10CA interactions deep within the pocket are highly similar to those of 6N-10CA and LRH-1 (Fig. 5, *C* and *D*) (47). The sulfamide moiety directly interacts with the side chains of T352, M268, and backbone of V326. It also engages a water-mediated hydrogen bond network with H310, R313, and L324 (Fig. 5*C*). The mediatory water has been modeled in previous structures of LRH-1 bound to 6N (44) and 6N-10CA (47) and was visible in omit maps in both chain A and B at σ = ∼4 and σ = ∼3, respectively. These are all interactions made by the sulfamide group with LRH-1. However, in LRH-1, 6N-10CA engages the backbone of phenylalanine (F404, LRH-1 numbering) rather than leucine (L324, SF-1 numbering) as well as the nearby methionine noted earlier (M345, LRH-1 numbering). The DPPE molecules in chains A and B have the same orientation as PLs complexed with SF-1 in previous structures, with the phosphates coordinated by Y436, K440, and the backbone of G341 (Fig. S2*C*). The strong density for DPPE interferes with proper refinement of the carboxylic acid tail position, pulling it away from the residues that coordinate this moiety in LRH-1 (Fig. 5, *C* and *D*). These residues correspond to phosphate-coordinating residues in SF-1 (Fig. 1*B*): G341, Y436, K440.

### Mutational analysis of residue contacts

**Figure 6:**
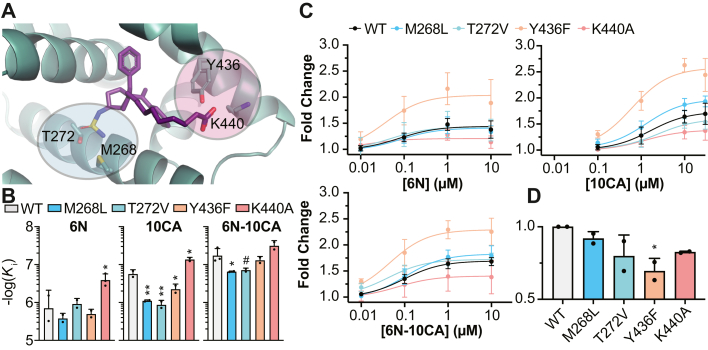
**Deep pocket and pocket mouth residues are responsible for small molecule binding and agonism.***A*, residues mutated for FP competition and luciferase reporter analyses in (*B*–*D*) are indicated. *B*, FP competition assay was used to assess the effect of indicated mutations on ligand binding. *K*_i_ = inhibition constant. Data shown as means + SD. Data compared to wild-type (WT) control: One-Way ANOVA with Dunnett multiple comparison’s test, #*p* < 0.1, ∗*p* < 0.05, ∗∗*p* < 0.01. *C*, luciferase reporter assays were used to assess the effect of indicated mutations on ligand-mediated activation of SF-1. Data were normalized relative to DMSO control and are shown as means ± SD from three biological replicates. *D*, luciferase reporter assay showing effects of mutations on basal SF-1 activity. Data normalized and analyzed relative to WT SF-1 activity and shown as means + SD from two biological replicates: One-Way ANOVA with Dunnett multiple comparison’s test, ∗*p* < 0.05.

We mutated residues that made direct contact with 6N-10CA deep within the pocket (M268 and T272) and at the mouth (Y436 and K440) to explore their roles in ligand binding and activation (Fig. 6*A*). Deep pocket mutations decreased SF-1 binding affinity for both 10CA and 6N-10CA (Fig. 6*B*), suggesting that ligands are sensitive to disruption of water-mediated and direct contacts deep in the pocket. Mutation of pocket mouth residue Y436 had minimal impact on 6N and 6N-10CA binding but decreased 10CA binding affinity, indicating that the sulfamide anchoring group can counteract reduced mouth pocket contacts. The K440A mutation consistently increased affinity of each agonist, although the increased affinity for 6N-10CA was statistically insignificant. This potentially highlights that this lysine is dispensable for ligand binding.

Examination of these mutations in reporter assays revealed that deep pocket mutations had little impact on compound potency or efficacy (Fig. 6*C*). However, the Y436F pocket mouth mutation consistently increased ligand-mediated activation. This mutant demonstrated diminished basal activity (Fig. 6*D*), presumably due to decreased capacity for PL ligand binding, which may have driven the greater fold changes observed. In contrast, the K440A mutation, while consistently improving compound binding, diminished efficacy of each small molecule, reflecting an important role that this residue plays for communicating ligand binding status. This indicates that the two critical residues at the SF-1 pocket mouth have distinct and important roles for compound binding and efficacy and that these roles are not limited to small molecules that mimic phospholipid contacts.

### MD analysis of ligand contacts

**Figure 7:**
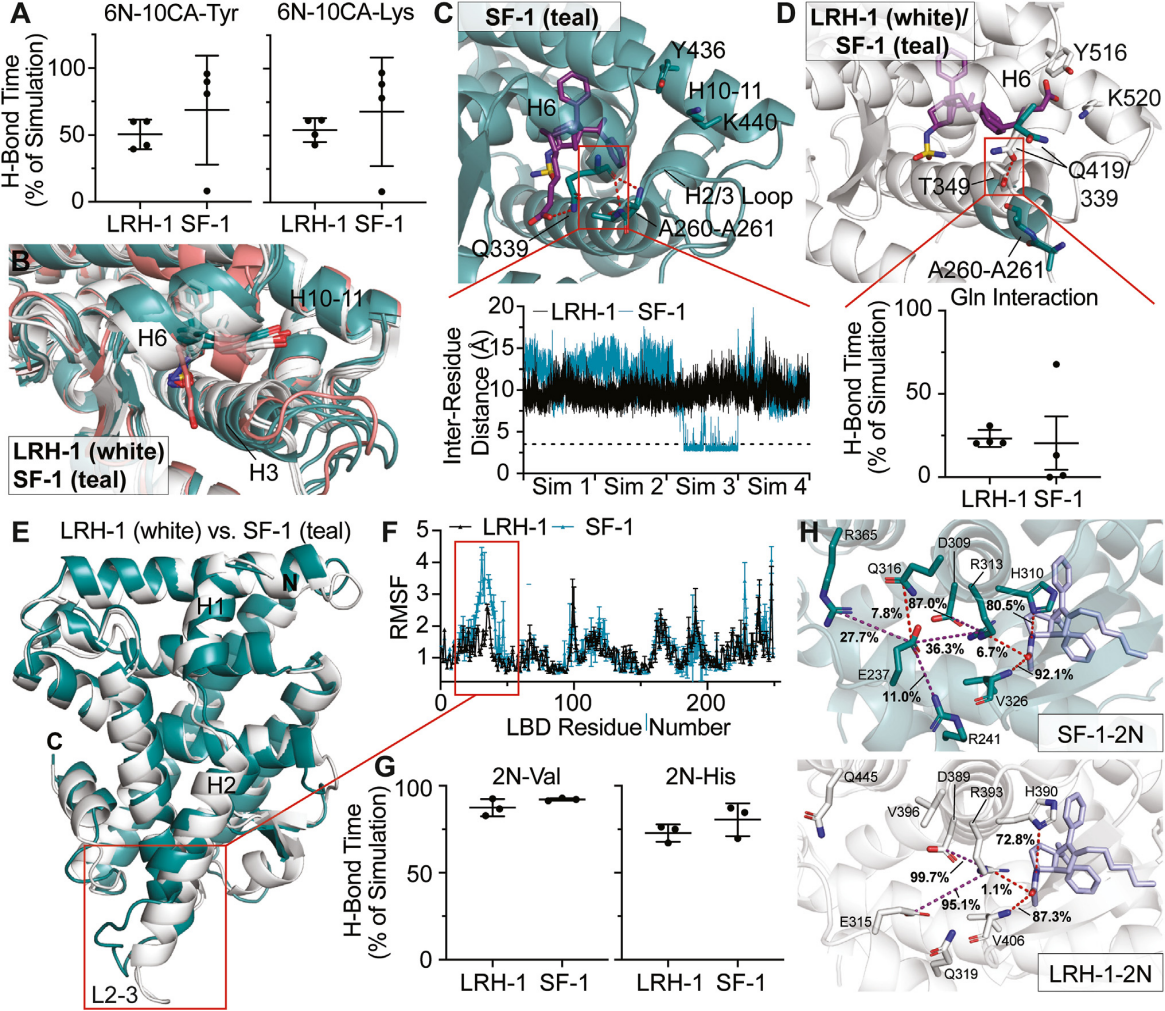
**MD simulations reveal differences in pocket mouth dynamics and small molecule binding.***A*, percentage of time that 6N-10CA spent in hydrogen bond with mouth residues Y516 (LRH-1)/Y436 (SF-1) and K520 (LRH-1)/K440 (SF-1) during MD simulations. Data shown as means ± SD from four 500-ns simulations. *B*, average atomic positions of 6N-10CA bound to LRH-1 (*white*) and SF-1 (*teal*). All four simulations are shown independently, with the repositioned 6N-10CA (and corresponding SF-1 LBD) shown in pink. *C*, *top*: Representative image of hydrogen bond contact between SF-1 L2-3 and helix 6. Bottom: Inter-residue distance between L2-3 alanine and helix 6 glutamine for LRH-1 (*black*) and SF-1 (*blue*) complexes. The *dotted line* corresponds to 3.5 Å. *D*, *top*: Representative image of inter-helical hydrogen bond in starting complex of LRH-1 (*white*). Corresponding residues on SF-1 are shown in *blue*. *Bottom*: Percentage of time that 6N-10CA spent in hydrogen bond with mouth residues Y516 (LRH-1)/Y436 (SF-1) and K520 (LRH-1)/K440 (SF-1) during MD simulations. The outlier seen in SF-1 corresponds to the simulation where the loop was repositioned (see panels (*A*–*C*)). *E*, LBD of LRH-1 (*white*) and SF-1 (*teal*) prior to MD simulations. L2-3 is highlighted with a *red box*. *F*, RMSF analysis of LRH-1-6N-10CA and SF-1-6N-10CA. Data represented as means ± SD from four 500-ns simulations. *G*, percentage of time that 2N spent in hydrogen bond with H390 (LRH-1)/H310 (SF-1) and V406 (LRH-1)/V326 (SF-1) during MD simulations. Data shown as means ± SD from three 500-ns simulations. *H*, representative images of critical deep pocket interactions from SF-1-2N (*top*) and LRH-1-2N (*bottom*) at the start of MD simulations. Important hydrogen bonds and electrostatic interactions are represented by red and purple dotted lines, respectively. Interaction duration is represented as the percentage of frames over three simulations.

We used MD simulations (4 × 500-ns) to compare ligand dynamics in SF-1 and LRH-1. While the starting positioning of the 6N-10CA tail was considerably different between LRH-1 and SF-1 complexes (Fig. 5*D*), the carboxylic acid quickly made and maintained hydrogen bond contacts with tyrosine and lysine residues at the mouth of the pocket for three of the four simulations (Fig. 7*A*). We noticed that the tail moved radically out of position early in one of the simulations (Fig. 7, *A* and *B*), and closer examination revealed that an inter-helical hydrogen bond was responsible for trapping the carboxylic acid in an orientation away from coordinating atoms at the pocket mouth (Fig. 7*C*). Dynamics of LRH-1 in this region is restricted by an inter-helical hydrogen bond driven by a threonine absent in SF-1 (Fig. 7*D*) and a longer helix 2 (Fig. 7*E*) observed in previously solved structures of the LBD, which results in a smaller root mean square fluctuation (RMSF) of corresponding LRH-1 residues (Fig. 7*F*) (32, 34, 35, 37, 41, 47, 52, 55). Therefore, as seen in LRH-1, 6N-10CA is capable of strong engagement with SF-1 residues at the mouth of the pocket, but can be repositioned away from these residues due to motions at the pocket mouth that are unique to SF-1.

Simulations (3 × 500-ns) with LRH-1-specific 2N revealed that this small molecule directly engages the backbone of a valine (LRH-1 V326; SF-1 V406) for nearly the entire simulation in both LRH-1 and SF-1 (Fig. 7*G*). Initial simulations indicated that 2N had substantially reduced hydrogen bonding with the deep pocket histidine (LRH-1 H390; SF-1 H310) in SF-1 complexes (Fig. S3). However, we found that this was driven by different starting rotamers of a nearby arginine (LRH-1 R393; SF-1 R313) (Fig. S3). Simulations with similar starting positions for this arginine indicated that 2N engaged in hydrogen bonds with both the nearby valine and histidine for comparable durations as LRH-1 simulations (Fig. 7*G*). Nevertheless, we noticed that this arginine is stabilized by an additional salt bridge with a nearby glutamate in LRH-1 for considerably longer than in SF-1 (Fig. 7*H*). This is likely driven by the presence of several nearby residues unique to SF-1 that compete for this side chain (Fig. 7*H*). It is possible that at longer time scales, this results in greater propensity for repositioning of R313 in SF-1, consequently destabilizing local contacts with the nearby histidine. This may explain why 2N shows specificity for LRH-1 in binding (38) and activity (44).

### MD analysis of ligand-driven allostery

**Figure 8:**
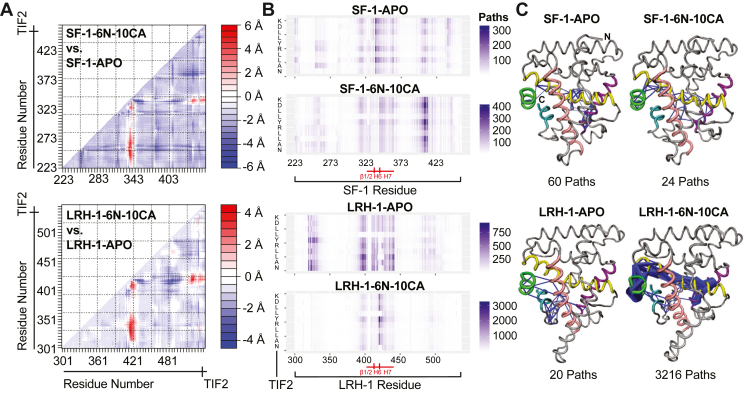
**6N-10CA drives stronger AF-B allosteric communication in LRH-1.***A*, all-residue (Cα) difference distance analysis of structures averaged after three 500-ns simulations. Inter-residue distances (Å) were determined for complexes and difference distance matrices were then calculated by subtracting distances from two complexes. The resulting matrices reveal structural differences between indicated complexes. *B*, matrices showing a number of paths between all residues on LRH-1-LBD and SF-1-LBD and their corresponding TIF2 peptides. Residues corresponding to the TIF2 peptide are on the vertical axis. Residues corresponding to SF-1 or LRH-1 are on the horizontal axis. Indicated in *red* are the regions that roughly correspond to the AF-B (β1/2-H6-H7). *C*, pathway analysis of LRH-1 or SF-1 LBD bound to ligands is shown (H3 = *pink*, H4/H5 = *yellow*, H7 = *purple*, H12 = *cyan*, TIF2 coactivator peptide = *green*). Paths between AF-B and TIF2 peptide are represented by *blue lines*. The number of paths between two nodes (Cα) is represented by edge weight. Note the SF-1-6N-10CA simulations were run with reoriented R313 (described in Fig. S3).

Activating ligands drive a conformation on the activation function surface (AF-2) on the LBD that promotes coactivator association. Coactivators drive target gene expression by recruiting transcriptional machinery and chromatin remodeling factors to gene loci (4). Previous simulations, paired with hydrogen-deuterium exchange mass spectrometry, have highlighted the importance of a region corresponding to β1/2, H6, and H7 on LRH-1 as an alternative activation function surface (AF-B) that communicates ligand-binding status to the AF-2 (35, 55). We sought to similarly use simulations (3 × 500-ns) to explore whether this region also plays a critical role in SF-1 activity. A comparison of average structures revealed that the AF-B is mobilized by 6N-10CA in both LRH-1 and SF-1 complexes (Fig. 8*A*). To explore allosteric paths of communication driven by ligands, we conducted suboptimal paths analysis. This analysis identifies correlated motion within chains of residues, which reflects allosteric communication between two distant sites on the protein (56, 57, 58). This has previously detected allosteric patterns that are correlated with activity of different ligand-bound states of LRH-1 (44, 47, 55, 59). We examined paths between all residues on the LBD and all residues on the co-complexed peptide fragment of coactivator TIF2 (Fig. 8*B*). Relative to the apo state, 6N-10CA dramatically increased paths of communication in LRH-1 between the region corresponding to the loop between helix 6 and 7 (“L6-7”) and TIF2. However, the SF-1 L6-7 was comparatively insensitive to 6N-10CA. Overlaying the paths between the glycine situated on this loop (G421, LRH-1 numbering; G341, SF-1 numbering) and TIF2 revealed that the effects of activating ligands are considerably more subtle in SF-1 (Fig. 8*C*). The number of paths and directionality of those paths is much more divergent between apo and ligand-bound states in LRH-1. This result suggests that while pocket mouth-contacting ligands such as 6N-10CA mobilize the AF-B region of both receptors, this region is not as critical for communicating ligand binding status for SF-1.

## Discussion

Here, we explored SF-1 activity using several small molecules that have been designed to enhance the activity of its close homolog LRH-1 (30, 44, 46, 47). Lead agonists 6N, 10CA, and 6N-10CA were able to bind and activate SF-1, with 6N-10CA showing the greatest affinity. As evidenced by this cross-reactivity with SF-1 and the nearly identical ligand binding pockets, one of the most challenging tasks for NR5A small molecule agonist development has been specifically targeting one NR5A. Here, we show numerous compounds that specifically activate LRH-1. Appending a 10CA tail to these molecules increases LRH-1 activity without promoting SF-1 cross-reactivity, providing useful tools for targeting LRH-1 where activation of SF-1 is undesirable.

It has been challenging to improve SF-1 modulators without the mechanistic insight provided by an SF-1-small molecule structure. However, the high affinity and stabilization provided by 6N-10CA enabled the solution of the first SF-1 crystal structure with any non-PL ligand. This structure showed co-occupancy with a bacterial phospholipid. Despite the incomplete ligand exchange, the SF-1-6N-10CA structure yielded valuable information about the molecular interactions between this agonist class and SF-1. The interactions in the pocket were nearly identical to those of 6N and 6N-10CA with LRH-1, with the sulfamide moiety forming an extensive hydrogen bond network with M268, H310, R313, L324, and V326 (44, 47). Only absent was the interaction with a nearby methionine (M345, LRH-1 numbering), as this residue is leucine in SF-1. While deep pocket interactions were nearly identical to those of LRH-1, the electron density of the bacterial PL hindered proper refinement of the 10CA tail.

We then explored how mutating residues directly contacted by 6N-10CA affected agonist binding and efficacy. We found that while deep pocket mutations slightly decrease the binding of carboxylic acid-containing small molecules, these mutations have minimal effects on activation. In contrast, pocket mouth mutations consistently either decreased (Y436F) or increased (K440A) binding affinity of small molecules. These mutations had the opposite effect on activity, with Y436F and K440A increasing and decreasing small molecule efficacy, respectively. The effect of Y436F may be the result of decreased basal activity in cells, which is likely driven by diminished hydrogen bond contact with activating PLs. The effect of K440A is more puzzling, as the mutation improved binding but decreased efficacy in reporter assays. This may be the consequence of the alanine mutation destabilizing allosteric motions at the pocket mouth while permitting increased access to the pocket interior. Altogether, these data suggest that while Y436 is critical for small molecule binding, K440 is important for communicating ligand binding status.

While the crystal structure confirmed a ligand orientation similar to that of LRH-1, it did not explain why 6HP-derived ligands consistently show greater affinity for LRH-1. This is the case even with 6N-10CA, which has an affinity for SF-1 over two orders of magnitude lower than for LRH-1 (Fig. 3*C* and reference (47)). We used MD simulations to elucidate how these small molecules show preference for LRH-1. While 6N-10CA makes stable contact with Y436 and K440, 25% of simulations show radical repositioning of the tail. LRH-1 has a long H2 and an H3-H6 interhelical bond which restricts local motion, while SF-1 is considerably more mobile due decreased helical character in this region. We note that this flexibility can result in closure of the SF-1 pocket mouth, trapping the hydrocarbon tail in an orientation away from the mouth residues. Interestingly, the same glutamine on H6 is responsible for the different inter-helical interactions visible in LRH-1 and SF-1 (Fig. 7, *C* and *D*). Previous studies have established that this loop (“L2-3”) on SF-1 is responsive to ligand-binding status. The headgroups of PIP2 and PIP3 promote stabilization of SF-1 L2-3 (37) and recent work by Bryant *et al.* showed that the acyl chain composition of PLs also influences order of L2-3 in crystal structures, which correlates with coactivator binding (41). This site can therefore sense favorable contacts at the pocket mouth and pocket interior. As was noted in this study (41), there are several mutations on L2-3 that have clinical significance, indicating that this site is critical for normative SF-1 activity (60, 61, 62, 63). Therefore, L2-3 appears to be important for PL-sensing, and this region is stabilized by the large head group of PIP3. This, along with the propensity for our ligands to reposition in this region, suggests that synthesizing 10CA variants with larger, PIP3-like moieties is an attractive route for improved SF-1 binding and agonism. We predict that larger moieties would prevent repositioning that is permitted by the small carboxylic acid tail in 6N-10CA and hydrocarbon tail on other ligands tested here.

While we identified several LRH-1-specific small molecules, the molecular basis for their specificity is not clear. The pocket interiors of LRH-1 and SF-1 are nearly identical, and the introduction of the nearby methionine (M345 in LRH-1) into the SF-1 pocket does not sensitize the receptor to 2N-10CA. While differences in pocket mouth motions may decrease the binding of ligands with a 10CA tail, these motions likely have a smaller effect on ligands such as 2N, which primarily contact deep pocket residues. A deeper investigation of MD simulations revealed that while residues proximal to small molecules are highly similar, those that lie directly outside of the binding pocket are not. We found that the positioning of the nearby arginine (R313 in SF-1) dramatically affects deep pocket interactions. This arginine is stabilized by a glutamate in LRH-1 (E315) for nearly the entire simulation. The corresponding glutamate in SF-1 (E237) interacts with R313 for a considerably shorter duration because of its propensity to engage with other interacting residues (*e.g.*, R241, Q316, and R365) not found in LRH-1. It is possible that at longer timescales, this results in destabilized deep pocket interactions. Therefore, we hypothesize that the molecular bases for LRH-1 specificity are different pocket-mouth dynamics and destabilized deep pocket interactions.

While we did not identify small molecules that showed specificity for SF-1, our findings suggest ideas for future designs. The rigid pocket mouth of LRH-1 may be incompatible with small molecules with inflexible tails and/or certain large polar moieties. Additionally, the small molecules tested here are racemic mixtures. Our recent investigations found critical differences in binding between enantiomers of parent compound RJW100 (54). Interestingly, while LRH-1 showed a clear preference for one enantiomer in reporter assays, SF-1 showed similar sensitivity to both enantiomers. Therefore, chiral separation may result in small molecules that are specific for SF-1.

MD simulations also revealed that 6N-10CA promotes mobility of the region corresponding to β1/2-H6-H7 in both SF-1 and LRH-1. This site is proximal to the binding pocket and includes a glycine on L6-7 that makes direct contact with the carboxylic acid tail on synthetic ligands, as well as the polar head group of PLs (Figs. 1*B* and 2*C*). This region (“AF-B”) was originally identified as a site on LRH-1 critical for communication driven by activating PL DLPC (35). More recent studies have identified this site’s role in differentiating coactivator and ligand identity (30, 44, 47, 54, 59). Pathway analysis revealed strong communication between this region and the TIF2 coactivator in LRH-1. However, 6N-10CA drove considerably weaker communication between L6-7 and TIF2 in SF-1, suggesting that this region may play less of a role for SF-1 activity. More direct methods, such as hydrogen-deuterium exchange mass spectrometry, are necessary to further explore ligand-driven allosteric communication in SF-1. Our simulations thus reveal different roles for both loops at the pocket mouth in ligand differentiation by NR5As.

With a highly effective agonist and the first synthetic small molecule-bound crystal structure of SF-1 in hand, new avenues of therapeutic development are now feasible. 6N-10CA would be a useful tool for elucidating SF-1’s role in altering gene expression in the VMH. These studies may also enable improvements in agonist efficacy. 6N-10CA has a potency nearly two times less and an affinity two orders of magnitude lower than LRH-1, indicating differences in the mechanism of activation and room for improvement toward SF-1 (47). The ability to directly compare SF-1- and LRH-1-6N-10CA structure and dynamics also offers the potential to develop compounds that specifically activate LRH-1, as noted above. While global activation of LRH-1 is unlikely to be detrimental, unnecessary activation of SF-1 in the adrenal glands may lead to the overproduction of adrenal steroids. Thus, our studies provide useful information and tools for LRH-1 agonism in the settings of diabetes, obesity, and inflammatory bowel disease. Finally, SF-1 antagonists are sought for the treatment of adrenocortical cancer, and this structure may yield strategies to modify 6N-10CA for receptor destabilization and antagonism. We note that both receptors are sensitive to the removal of the R^2^ group, which is positioned proximal to the AF-2. Modification of this moiety may drive NR5A inverse agonism, and this is currently being pursued in our lab. Altogether, our studies offer novel insight into the mechanisms underlying ligand-mediated activation of SF-1 and provide useful tools for specifically targeting its close homolog LRH-1.

## Experimental procedures

### Chemical synthesis

Synthesis of 6N-10CA has been previously reported (38, 47). Synthetic methods for 1-7N have also been previously published along with RJW100 (44). The carboxylic acid derivate (10CA) was synthesized as previously described (46). Synthesis of 6N-10CA (No R^2^), 2N-10CA, and 4N-10CA are detailed in the supplemental information. Note that while all compounds tested in these studies are racemic mixtures, the enantiomers used for figures and MD simulations are based on evidence from previous crystal structures (44, 47).

### Cell culture

HeLa cells were cultured under standard conditions (5% CO_2_, 37°C) in phenol red-free MEMα + 10% fetal bovine serum (FBS) – charcoal/dextran treated and were verified to be *mycoplasma* free with the LookOut *Mycoplasma* PCR Detection Kit.

### Protein purification of wild-type SF-1

*E. coli* strain BL21(DE3)-pLysS was transformed with the SF-1 LBD (amino acids 218–461) in the pLIC-His vector and cultured at 37 °C to OD_600_ of 0.6 in Lysogeny Broth medium in the presence of chloramphenicol and ampicillin. Protein expression was induced with 0.5 mM isopropyl-1-thio-D-galactopyranoside for 4 h at 32 °C. Cell pellets were lysed in 125 ml NiA (500 mM NaCl, 25 mM imidazole, 5% glycerol, 20 mM Tris HCl pH 7.4, 0.5 mM TCEP) with lysozyme, phenylmethylsulfonyl fluoride, and DNase followed by sonication. Lysate was clarified by centrifugation in a Sorvall RC 6+ centrifuge at 16,000*g* for 45 min. Supernatant was flowed over a 5 ml HisTrap FF column (GE Healthcare) and protein was eluted with NiB (NiA with 500 mM imidazole). To homogenize the lipid population, SF-1 was incubated overnight with DLPC. Size exclusion chromatography (SEC) into assay buffer (150 mM NaCl, 20 mM Tris HCl pH 7.4, 5% glycerol) was used as a final purification step. Protein was concentrated to ∼3 mg/ml, flash frozen, and stored at −80 °C for use in assays.

### Protein purification of CysLite SF-1

CysLite SF-1 (amino acids 218–461, C247S, C412S) in the pLic-His vector was used for crystallization. This protein was purified as described for wildtype SF-1 through the HisTrap column; after elution from the HisTrap column, the 6X-His tag was cleaved overnight using tobacco etch virus protease. Cleaved protein was flowed over a second HisTrap column and the flowthrough was collected, concentrated to ∼3 mg/ml, flash frozen, and stored at −80 °C for use in crystallization.

### Differential scanning fluorimetry

Purified SF-1 LBD, pre-exchanged with DLPC (0.2 mg/ml), was combined with small molecules overnight at 4 °C in assay buffer. SYPRO orange dye was added to the complexes the next day, at a final dilution of 1:1000. Complexes were heated at a rate of 0.5 °C/minute on a StepOne Plus thermocycler, using the ROX filter for fluorescence detection. The melting temperature (Tm, 50% unfolding) was calculated using the Bolzman equation GraphPad Prism (version 9). Assays were conducted with nine technical replicates over three experiments.

### Fluorescence polarization

FP assays were performed as described previously (38, 47, 53). Briefly, experiments were conducted in black, polystyrene, non-binding surface 384-well plates (Corning Inc., Corning, NY) with 30 μl volumes in assay buffer (150 mM NaCl, 20 mM Tris-HCl, 5% glycerol, pH 7.4). Binding affinity for 6N-FAM was determined using 10 nM 6N-FAM and protein concentrations ranging from 1^-10^ - 5^-5^ M. Plates were incubated overnight at 4 °C and centrifuged at 2000*g* for 2 min before polarization measurement. Polarization was monitored on a Neo plate reader (Biotek, Winooski, VT) at an excitation/emission wavelength of 485/528 nm. Plates were incubated overnight at 4 °C and centrifuged at 2000*g* for 2 min before polarization measurement. Nine technical replicates were conducted over three experiments and compiled binding data were baseline-corrected to wells with no protein and fit with a one-site binding curve in GraphPad Prism version 7 (GraphPad, Inc, La Jolla, CA). 6N conjugated to fluorescein amidite (FAM) (10 nM/well – 0.8 times the affinity of SF-1 for 6N-FAM) was incubated with SF-1 LBD (25 nM/well – 60% of the forward binding B_max_). Unlabeled compounds were added at concentrations indicated in figures with DMSO in each well held constant at 6.7% v/v. Each experiment was performed two or three times with four technical replicates each. Technical replicates were averaged and normalized independently prior to final data analysis. Using GraphPad Prism (version 9), data were fit to a one-site, fit *K*_i_ curve, assuming a final probe concentration of 10 nM and probe affinity determined with forward binding assays (WT: 12 nM, M268L: 19.3 nM, T272V: 10.9 nM, Y436F: 9.9 nM, K440A: 5.5 nM).

### Luciferase reporter

Reporter assays were conducted as described previously (47, 52, 53). Briefly, HeLa cells were seeded at ∼7500 to 10,000 cells per well in 96-well plates (white-walled, clear bottom) in MEMα + 10% FBS – charcoal/dextran treated. Once cells reached 80 to 90% confluence, they were transfected with LRH-1 (in pCI vector, 5 ng/well) or SF-1 (in pcDNA vector, 5 ng/well), a reporter plasmid with an *NR5A* response element derived from the *SHP* promoter cloned upstream of *Firefly* Luciferase (in pGL3-Basic vector, 50 ng/well), and a plasmid expressing *Renilla* Luciferase constitutively from a *CMV* promoter (1 ng/well). Cells were transfected with FuGENE at a ratio of 3:1 (FuGENE:DNA). Twenty-four hours after transfection, compounds were diluted in Opti-MEM and introduced to cells at the final concentrations indicated in figures (final DMSO concentration was 0.37%). Luciferase signal was measured after ∼24 h using the DualGlo kit (Promega) with a BioTek Neo plate reader. Each experiment was conducted with three biological replicates, each with three technical replicates averaged prior to data analysis. Firefly Luciferase signal for each well was divided by the well’s Renilla Luciferase signal intensity and then normalized relative to the DMSO control. Data were analyzed with GraphPad Prism (version 9) using a stimulating dose–response curve (Hill slope = 1). Data were excluded from analysis for cells treated with 3e-5 M of 6N and 6N-10CA, as the final signal showed a drastic decrease in overall signal, potentially indicating cell toxicity or compound insolubility.

### Sequence alignment

Sequences were aligned with Clustal Omega (64, 65, 66).

### Crystallization

Cleaved SF-1 CysLite was incubated overnight with 4-fold molar excess compound 6N, and SEC was used to remove bacterial lipids and excess compound and exchange into crystallization buffer (150 mM NaCl, 100 mM ammonium acetate (pH 7.4), 1 mM DTT, 1 mM EDTA, 2 mM 3-[(3-cholamidopropyl)dimethylammonio]-1propanesulfonic acid (CHAPS)). An additional 2-fold molar excess of 6N was added to SF-1 collected after SEC. SF-1 was concentrated to ∼5 mg/ml, 4-fold molar excess of the TIF2 peptide (^+^H_3_N-KENALLRYLLDKDD-CO_2_^-^) was added, and the complex was incubated at room temperature for 2 h. Crystals were seeded with LRH-1-RJW100 crystals, grown as previously described (52). Crystals were grown using hanging drop vapor diffusion at 4 °C with 0.05 mM Na acetate (pH 4.6), 0 to 25% glycerol, and 5 to 11% PEG 4000, with 2 to 4 μl drops. The crystals contained bacterial phospholipid rather than 6N. Thus, soaking of 6N-10CA, which had a considerably higher affinity than 6N, was used to exchange the phospholipids. 6N-10CA (100 mM DMSO) was diluted to 2.5 mM in mother liquor, and 0.5 μl volumes were added to drops containing crystals for a 2-days soak. Crystals were flash-frozen in liquid nitrogen using mother liquor with a cryoprotectant of 30% glycerol.

### Structure determination

Data were collected remotely from the Southeast Regional Collaborative Access Team (SER-CAT) at the Advanced Photon Source (Argonne National Laboratories) using the 22ID beamline. Data were processed and scaled using HKL2000 (67) and phased by molecular replacement using Phaser-MR in Phenix (68) with PDB entry 1ZDT as the search model. Coot (69) and Phenix.refine (68, 70) were used for model building and refinement, respectively. Additional refinement was performed with PDB Redo (71). Figures were constructed using Pymol (72). Hydrogen bonds in all figures were shown if indicated atoms were within ∼3.5 Å of one another unless otherwise indicated. Omit maps were generated by removing all ligand atoms from the model and running an additional round of refinement.

### Mutagenesis

All mutations other than L265M were introduced using the QuikChange II Site-Directed Mutagenesis Kit (Agilent). The Q5 Site-Directed Mutagenesis Kit (New England Biolabs) was used to generate the L265M mutant. All constructs were sequenced prior to use.

### MD simulations

MD simulations were conducted similarly to previous studies (47). LRH-1 and SF-1 models were prepared using SF-1-TIF2-6N-10CA (chain B of PDB entry 8DAF) and LRH-1-TIF2-6N-10CA (PDB entry 7TT8) as starting structures. For SF-1 complexes, PDB entry 4QK4 (residues 239–265) was used to replace missing residues. SF-1 residues 221 to 222 and TIF2 residue 741 were removed to keep LRH-1 and SF-1 complexes consistent. Surface cysteines mutated to serine for crystallography were mutated back to cysteine for simulations. For LRH-1 complexes, PDB entry 4PLE (residues 321–345) was used to replace missing residues. LRH-1 residues 299 to 300 were removed to keep LRH-1 and SF-1 complexes consistent.

For 6N-10CA-bound structures, PDB entry 8DAF (chain B) and PDB entry 7TT8 were used as starting models. For 2N-bound constructs, the position of 2N from the previously solved crystal structure of LRH-1-2N (PDB entry 6OR1) was used to guide the ligand into the pocket of LRH-1 and SF-1. Although the density in the crystal structure was satisfied by various orientations for R313, initial simulations indicated differences in histidine interactions driven by side chain orientation. Therefore, we repeated SF-1-2N simulations with this residue reoriented to better reflect the R393 rotamer in LRH-1 complexes (see Fig. S3 for representative images). Note that we also repositioned R313 in the SF-1-6N-10CA complex used for average structure comparison and pathway analyses (Fig. 8) so that the starting deep pocket interactions in LRH-1 and SF-1 complexes were similar. Apo complexes were generated by removing 6N-10CA from the prepared LRH-1- and SF-1-6N-10CA complexes used in initial MD simulations. Maestro (Schrödinger, LLC) (73) was used to optimize hydrogen bond assignments, add N- and C-terminal caps to LRH-1, SF-1, and TIF2, and run initial minimization on the structure. The complexes were then solvated in an octahedral box of TIP3P water with a 10 Å buffer around the protein complex. Na^+^ and Cl^−^ ions were added to neutralize the protein and achieve physiological buffer conditions (150 mM NaCl). Parameters for 6N, 10CA, and 6N-10CA were obtained using Antechamber (74) in AmberTools20. Systems were set up using the xleap tool in AmberTools20 of Amber 2020 (75), with ff14SB (76) (protein), GAFF2 (77) (ligand), and TIP3P (78) (water) forcefields.

Minimizations and simulations were performed with Amber20. For minimization, 5000 steps of steepest descent were used, followed by 5000 steps of conjugate gradient minimization. Minimizations were first performed with 500 kcal/mol·Å2 restraints on all protein and ligand atoms. Restraints were then removed on all atoms except the ligand and TIF2 peptides, and the protocol was repeated. Restraints were then removed from TIF2 atoms, and the protocol was repeated. Ligand restraints were then lowered to 100 kcal/mol·Å2 for an additional round of minimization. Restraints were then removed from all atoms for two final rounds of minimization. Minimized systems were heated from 0 to 300 K with a 100 ps MD run, with constant volume periodic boundaries and 10 kcal/mol·Å2 restraints on all protein and ligand atoms. A 10 ns equilibration was then performed with 10 kcal/mol·Å2 restraints on all protein and ligand atoms. Restraints were then lowered to 1 kcal/mol·Å2 for ligand and protein atoms and a 10 ns equilibration was performed. A final 10 ns round of equilibration was then run with only ligand atoms restrained (1 kcal/mol·Å2). Note that the water molecule critical for ligand engagement deep within the pocket was restrained along with ligand atoms. Finally, production trajectories of 500 ns were obtained for unrestrained complexes in the NPT ensemble. All bonds between heavy atoms and hydrogens were fixed with the SHAKE algorithm (79). A cutoff distance of 10 Å was used to evaluate long-range electrostatics with particle mesh Ewald and for van der Waals forces. Three (2N-bound and apo complexes, as well as the 6N-10CA-bound SF-1 complex prepared for pathway analysis) or four (6N-10CA-bound LRH-1 complexes and SF-1-6N-10CA used for initial ligand binding analysis) 500-ns simulations were run. Frames from MD simulations were concatenated with CPPTRAJ (80) for comparison of averaged 6N-10CA-bound complexes, as well as pathway analysis. Every fifth frame was used for data analysis. We excluded one 500-ns simulation from LRH-1-6N-10CA average structure and pathway analyses to keep analysis consistent with SF-1 complexes. Water, Na^+^, and Cl^-^ molecules, along with N- and C-terminal caps, were removed for data analysis.

CPPTRAJ (80) was used for hydrogen bond, distance, and RMSF analyses as well as the construction of average structures. Note that hydrogen bond analysis was conducted with the default angle cutoff of 135° and distance cutoff of 3.5 Å. Frames with any interacting atoms with noted residues were counted in calculations. Note that only side chains were considered for Figure 7*H* (other than interaction with the nearby valine backbone). Bio3D (81) was used to create difference distance matrices comparing average structures between apo and 6N-10CA-bound complexes. Dynamic networks were constructed from trajectories using the NetworkView plugin (57) in VMD (82) and the Carma program (83). Networks are constructed by defining all protein Cα atoms as nodes, using Cartesian covariance (calculated in Carma) to measure communication within the network. Hydrogen atoms were excluded from network construction, and edges between neighboring residues were disallowed. Pairs of nodes that reside within a 4.5 Å cutoff for 75% of the simulation are connected *via* an edge. Suboptimal paths between nodes were identified using the Floyd−Warshall algorithm (84). Matrices visualizing the number of paths between every residue on LRH-1 or SF-1 and the corresponding TIF2 peptide was determined using a loop script previously described (47), using a cutoff value of 30 in the subopt executable file. Communication with residue Y_6_ on the TIF2 peptide (N_1_A_2_L_3_L_4_R_5_Y_6_L_7_L_8_D_9_K_10_) was used as the sink for pathway analysis, as this residue displayed strong communication with the AF-B in LRH-1-6N-10CA complexes. Suboptimal paths were visualized with VMD (82).

## Data availability

Crystal structure atomic coordinates and experimental data are available in the Protein Data Bank (PDB ID: 8DAF).

## Supporting information

This article contains supporting information (47, 53).

## Conflict of interest

The authors declare that they have no conflicts of interest with the contents of this article.
