# Supplementary material for: Comparison of activity, structure, and dynamics of SF-1 and LRH-1 complexed with small molecule modulators
Source: J Biol Chem. 2023 Jun 14;299(8):104921. doi: 10.1016/j.jbc.2023.104921 (PMC10407255; doi:10.1016/j.jbc.2023.104921)
Supplement: Supporting information [file mmc1.docx]

**SUPPORTING INFORMATION.**

**Figure S1. Introduction of methionine to SF-1 pocket does not sensitize receptor to LRH-1-selective compounds.** Luciferase reporter assays were used to examine whether L265M mutation in SF-1 confers sensitivity to LRH-1-selective small molecule 2N-10CA. Four bars for each ligand represent (from left to right) DMSO (control) and 2N-10CA at 0.1, 1, and 10 µM. Data shown as means + SD from three biological replicates. Data normalized relative to DMSO control.

**Table S1. X-ray data collection and refinement statistics.** Values in parentheses indicate highest resolution shell.

**Figure S2. Chain A density reveals dual occupancy.** **A.** When all ligands are omitted from the map, 6N-10CA and DPPE together satisfy the ligand density for chain A, but not 6N-10CA or DPPE alone. Omit maps are F_o_-F_c_ contoured at σ = 2.0. **B.** 6N-10CA for both chains are modeled with the same binding pose. **C.** The pocket mouth residues for both chains are in the same positioning and are oriented to make hydrogen bond contact with the phospholipid head. In white is a PL modeled in a previous structure of SF-1 complexed with DPPE (PDB entry 1ZDT). PDB entry 1ZDT side chains are also represented as white sticks.

**Figure S3. Position of arginine side chain affects deep pocket interactions with 2N.** Top: Starting positions of nearby side chains of interacting residues in SF-1 and LRH-1 complexes. R313 was repositioned to better mimic the starting position of R393 in LRH-1-2N. Bottom: Resulting hydrogen bonds and electrostatic interactions are represented by red and purple dotted lines, respectively. Interaction duration is represented as the percentage of frames over three simulations.

**CHEMISTRY**

All reactions were carried out in flame-dried glassware, equipped with a stir bar and under a nitrogen atmosphere with dry solvents under anhydrous conditions, unless otherwise noted. Solvents used in anhydrous reactions were purified by passing over activated alumina and storing under argon. Yields refer to chromatographically and spectroscopically (^1^H NMR) homogenous materials, unless otherwise stated. Reagents were purchased at the highest commercial quality and used without further purification, unless otherwise stated. n-Butyllithium (n-BuLi) was used as a 2.5 M solution in hexanes (Aldrich), was stored at 4°C and titrated prior to use. Organic solutions were concentrated under reduced pressure on a rotary evaporator using a water bath. Chromatographic purification of products was accomplished using forced-flow chromatography on 230-400 mesh silica gel. Thin-layer chromatography (TLC) was performed on 250μm SiliCycle silica gel F-254 plates. Visualization of the developed chromatogram was performed by fluorescence quenching or by staining using KMnO4, *p*-anisaldehyde, or ninhydrin stains.

^1^H and ^13^C NMR spectra were obtained from the Emory University NMR facility and recorded on a Bruker Avance III HD 600 equipped with cryo-probe (600 MHz), INOVA 600 (600 MHz), INOVA 500 (500 MHz), INOVA 400 (400 MHz), VNMR 400 (400 MHz), or Mercury 300 (300 MHz), and are internally referenced to residual protio solvent signals. Data for ^1^H NMR are reported as follows: chemical shift (ppm), multiplicity (s = singlet, d = doublet, t = triplet, q = quartet, m = multiplet, dd = doublet of doublets, dt = doublet of triplets, ddd= doublet of doublet of doublets, dtd= doublet of triplet of doublets, b = broad, etc.), coupling constant (Hz), integration, and assignment, when applicable. Data for decoupled ^13^C NMR are reported in terms of chemical shift and multiplicity when applicable. Liquid Chromatography Mass Spectrometry (LC-MS) was performed on an Agilent 6120 mass spectrometer with an Agilent 1220 Infinity liquid chromatography inlet. Preparative High-Pressure Liquid chromatography (Prep-HPLC) was performed on an Agilent 1200 Infinity Series chromatograph using an Agilent Prep-C18 30 x 250 mm 10 μm column, or an Agilent Prep-C18 21.2 x 100 mm, 5 μm column.

**10-((3a*R*,6*S*)-6-acetamido-3-phenyl-3a-(1-phenylvinyl)-1,3a,4,5,6,6a-hexahydropentalen-2-yl)decanoic acid** (**2N-10CA**): To a flame-dried reaction vial equipped with a stirbar backfilled to a nitrogen atmosphere was added 10-((3a*R*,6*S*)-6-amino-3-phenyl-3a-(1-phenylvinyl)-1,3a,4,5,6,6a-hexahydropentalen-2-yl)decanoate (prepared as previously reported)(1) (0.03 mmol, 1.0 equiv, 14.4 mg) in DCM (280 μL). The reaction vial was cooled to 0 °C before the dropwise addition of acetyl chloride (0.04 mmol, 1.5 equiv, 3.0 μL) and triethylamine (0.09 mmol, 3.0 equiv, 11.6 μL), respectively. The reaction stirred for 1 hour warming to room temperature before being quenched with water. The organic layer was then removed and the aqueous layer was reextracted (3x) with DCM. The combined organic layers were dried with NaSO_4_, filtered, and concentrated via rotary evaporation. The crude oil was redissolved in dioxanes (500 μL) with 2 drops of HCl. After 48 hours the reaction showed consumption of starting material. The reaction was then diluted in EtOAc and washed with water (2x). The organic layer was dried with NaSO_4_, filtered, and concentrated via rotary evaporation. The oil was purified via flash chromatography (50-100% EtOAc/Hexanes) to give the title compound (4.3 mg, 42% yield) as a clear oil.

**^1^H NMR** (600 MHz, cdcl_3_) δ 7.33 – 7.27 (m, 5H), 7.23 (dd, *J* = 7.2, 2.5 Hz, 5H), 5.37 (d, *J* = 8.1 Hz, 1H), 5.05 (d, *J* = 1.4 Hz, 1H), 5.01 (d, *J* = 1.5 Hz, 1H), 4.25 (dtd, *J* = 10.6, 8.5, 6.2 Hz, 1H), 2.66 (td, *J* = 8.6, 2.0 Hz, 1H), 2.29 (t, *J* = 7.5 Hz, 2H), 2.04 (tdd, *J* = 17.6, 7.8, 3.6 Hz, 3H), 1.98 (s, 3H), 1.86 (dtd, *J* = 11.8, 6.0, 2.3 Hz, 1H), 1.75 (td, *J* = 12.3, 5.9 Hz, 1H), 1.66 (td, *J* = 6.4, 2.3 Hz, 1H), 1.60 (t, *J* = 7.2 Hz, 2H), 1.35 (ddt, *J* = 16.7, 11.4, 5.7 Hz, 2H 3?), 1.31 – 1.20 (m, 13H).

**^13^C NMR** (151 MHz, CDCl_3_) δ 175.83, 169.75, 154.33, 143.47, 142.97, 139.31, 136.89, 129.84, 129.48, 128.85, 128.02, 127.82, 127.68, 127.57, 126.77, 114.99, 69.03, 53.26, 47.48, 35.25, 33.34, 32.06, 31.63, 29.97, 29.71, 29.68, 29.24, 29.20, 29.09, 28.96, 28.06, 24.67, 23.33, 22.70.

**LRMS** **(APCI)** m/z: [M+H]^+^ calc’d. for C_34_H_44_NO_3_: 514.7, found 514.4

**10-((3a*R*,6*S*)-3-phenyl-3a-(1-phenylvinyl)-6-ureido-1,3a,4,5,6,6a-hexahydropentalen-2-yl)decanoic acid** (**4N-10CA**): To a flame-dried reaction vial equipped with a stirbar backfilled to a nitrogen atmosphere was added 10-((3a*R*,6*S*)-6-amino-3-phenyl-3a-(1-phenylvinyl)-1,3a,4,5,6,6a-hexahydropentalen-2-yl)decanoate (0.04 mmol, 1.0 equiv, 20 mg) (prepared as previously reported)(1) dissolved in dry MeCN. Triethylamine (0.08 mmol, 2.0 equiv, 11 μL) was added followed by (trimethylsilyl)isocyanate (0.08 mmol, 2.0 equiv, 11 μL). The reaction proceeded overnight at ambient temperatures before being pushed through a silica plug and concentrated. The crude material was purified via flash chromatography (100% EtOAc). The crude oil was redissolved in a 1:1 mixture of H_2_O/THF (400 μL) with LiOH (0.06 mg, 3.0 equiv, 1.5 mg). After 16 hours the reaction showed consumption of starting material. The reaction was then diluted in EtOAc and washed with water (2x). The organic layer was dried with NaSO_4_, filtered, and concentrated via rotary evaporation. The oil was purified via flash chromatography (10% MeOH/DCM) to give the title compound (3.0 mg, 15% yield) as a clear oil.

**^1^H NMR** (500 MHz, cdcl_3_) δ 7.34 – 7.29 (m, 8H), 7.19 (d, *J* = 7.2 Hz, 2H), 5.09 (s, 1H), 4.92 (s, 1H), 4.60 (br. s, 1H), 2.52 (s, 1H), 2.33 (dt, *J* = 17.7, 9.5 Hz, 2H), 2.12 (dt, *J* = 15.2, 8.0 Hz, 1H), 2.04 – 1.88 (m, 2H), 1.77 – 1.66 (m, 4H), 1.59 (d, *J* = 7.4 Hz, 4H), 1.32 (d, *J* = 62.4 Hz, 13H).

**^13^C NMR** (151 MHz, CDCl_3_) δ 169.75, 154.33, 134.81, 129.78, 127.90, 127.73, 127.72, 126.77, 58.51, 50.91, 40.95, 37.11, 34.68, 33.71, 33.44, 32.49, 30.18, 30.05, 29.46, 29.38, 29.26, 29.09, 27.10, 26.72, 26.56, 25.91, 25.29, 24.76, 23.19, 22.71, 22.67, 20.71, 19.74.

**LRMS** **(APCI)** m/z: [M+H]^+^ calc’d. for C_33_H_43_N_2_O_3_: 515.7, found 515.3

**10-(6-oxo-3-phenyl-1,3a,4,5,6,6a-hexahydropentalen-2-yl)decanoic acid** (**S1**): To reaction vial equipped with a stirbar was added 10-(6-hydroxy-3-phenyl-1,3a,4,5,6,6a-hexahydropentalen-2-yl)decanoic acid (0.6 mmol, 1.0 equiv, 206 mg) (prepared as previously reported) (2) dissolved in DCM (11.2 mL). Dess-Martin Periodinane (0.7 mmol, 1.2 equiv, 284 mg) was added and the reaction proceeded overnight at ambient temperatures before being pushed through a silica plug. The filtrate was concentrated and the crude material was purified via flash chromatography (50% EtOAc/Hex) to give the title compound (154.4 mg, 75% yield) as a clear oil.

**^1^H NMR** (600 MHz, CDCl_3_) δ 7.38 (dd, *J* = 8.3, 6.9 Hz, 2H), 7.27 (m, 1H), 7.18 – 7.16 (m, 2H), 3.95 (d, *J* = 7.1 Hz, 1H), 2.38 – 2.34 (m, 3H), 2.20 (td, *J* = 7.1, 2.6 Hz, 2H), 2.18 – 1.97 (m, 3H), 1.85 (ddt, *J* = 13.0, 9.2, 1.7 Hz, 1H), 1.71 – 1.60 (m, 4H), 1.58 – 1.51 (m, 1H), 1.45 – 1.18 (m, 12H).

**^13^C NMR** (151 MHz, CDCl_3_) δ 141.04, 137.38, 137.03, 128.31, 128.22, 126.65, 68.11, 50.99, 48.82, 39.43, 36.17, 33.76, 29.41, 29.31, 29.26, 29.15, 29.01, 28.98, 28.88, 28.65, 28.43, 27.99, 24.67, 23.98.

**LRMS** **(APCI)** m/z: [M+H]^+^ calc’d. for C_24_H_33_O_3_: 369.5, found 369.3.

**methyl 10-(6-oxo-3-phenyl-1,3a,4,5,6,6a-hexahydropentalen-2-yl)decanoate** (**S2**): ): To reaction vial equipped with a stirbar was added **S1** (0.2 mmol, 1.0 equiv, 77.2 mg) dissolved in methanol (2 mL). Two drops of hydrochloric acid was added to the reaction and was stirred overnight at ambient temperatures before being concentrated. The crude material was purified via flash chromatography (5-10% EtOAc/Hex) to give the title compound (79.7 mg, 96% yield) as a clear oil.

**^1^H NMR** (600 MHz, CDCl_3_) δ 7.40 – 7.36 (m, 2H), 7.28 – 7.26 (m, 1H), 7.18 – 7.16 (m, 2H), 3.95 (t, *J* = 6.9 Hz, 1H), 3.68 (s, 3H), 2.84 – 2.65 (m, 2H), 2.31 (t, *J* = 7.6 Hz, 2H), 2.26 – 1.90 (m, 5H), 1.89 – 1.82 (m, 1H), 1.62 (p, *J* = 7.5 Hz, 2H), 1.38 (dt, *J* = 8.8, 6.0 Hz, 1H), 1.34 – 1.13 (m, 12H).

**^13^C NMR** (151 MHz, CDCl_3_) δ 174.35, 141.04, 137.36, 137.04, 128.31, 128.22, 126.65, 51.45, 50.98, 48.80, 39.43, 36.16, 34.11, 29.46, 29.33, 29.30, 29.21, 29.12, 28.01, 24.95, 23.98.

**LRMS** **(APCI)** m/z: [M+H]^+^ calc’d. for C_25_H_35_O_3_: 383.6, found 383.3.

**methyl 10-((6*S*)-6-amino-3-phenyl-1,3a,4,5,6,6a-hexahydropentalen-2-yl)decanoate** (**S3**): To a flame-dried reaction vial equipped with a stirbar backfilled to a nitrogen atmosphere was added **S2** (0.2 mmol, 1.0 equiv, 67.6 mg) dissolved in dry ethanol. Titanium (IV) isopropoxide (0.3 mmol, 1.5 equiv, 80.4 μL) was added followed by ammonia 7N in methanol (3.53 mmol, 20 equiv, 504 μL). The reaction proceeded for 6 hours at ambient temperatures, turning yellow, before the addition of sodium borohydride (0.53 mmol, 3 equiv, 20 mg) at 0 ^o^C. The reaction proceed overnight warming to room temperature before being poured into a separatory funnel. The mixture was partitioned between water and EtOAc. The organic layer was collected and washed with brine (2x). The crude material was pushed through a silica plug (10-50% EtOAc/Hex + 1% TEA then 100% EtOAc+ 1% TEA). The oil was then dissolved in dry benzene under nitrogen and was used without further purification in subsequent steps.

**10-((6*S*)-3-phenyl-6-(sulfamoylamino)-1,3a,4,5,6,6a-hexahydropentalen-2-yl)decanoic acid** (**6N-10CA (no R2)**): An oven-dried vial was charged with a stirbar, *^t^*BuOH (1.23 mmol, 91.7 mg), and DCM (11.2 ml) then evacuated under reduced pressure and backfilled with nitrogen three times and cooled to 0 ºC. Chlorosulfonyl isocyanate (1.125 mmol, 97 µl) was then added dropwise via syringe and the solution allowed to warm to 23 ºC over 90 minutes. A 1.63 ml portion of this solution was added slowly via syringe to a solution of **S3** (0.15 mmol, 1.0 equiv 57 mg) and triethylamine (0.297 mmol, 2.0 equiv, 41 µl) in DCM (1.47 ml) at 0 ºC under nitrogen. This combined solution was allowed to warm to 23 ºC gradually 16 h then diluted with EtOAc. The diluted solution was washed with three times with NH_4_Cl then H_2_O and brine. The organic layer was dried over Na_2_SO_4_, filtered, and filtrate concentrated under reduced pressure to collect the crude material. The crude material was dissolved in dioxane (508 µL). The solution was frozen in an ice bath and then allowed to slowly warm to 23 ºC. As soon was the entire solution had re-melted, cold concentrated HCl (169 µL) was added so the solution was 3:1 HCl: Dioxane. The solution was allowed to slowly warm to 23 ºC and continue reacting at 40 °C until starting material was consumed by TLC. The reaction solution was diluted with EtOAc and washed four times with H_2_O then twice with brine. The organic layer was dried over Na_2_SO_4_, filtered, and filtrate concentrated under reduced pressure to collect the crude material. This crude material was purified by flash chromatography on silica with 30-45% EtOAc/hexanes to collect the title compound (4.4 mg, 7% yield).

**^1^H NMR** (600 MHz, CDCl_3_) δ 7.26 (t, *J* = 7.6 Hz, 2H), 7.17 – 7.14 (m, 1H), 7.12 – 7.09 (m, 2H), 4.96 (d, *J* = 7.8 Hz, 1H), 4.55 (s, 2H), 3.76 (dtd, *J* = 10.2, 7.8, 5.5 Hz, 1H), 3.55 (t, *J* = 8.7 Hz, 1H), 2.97 – 2.90 (m, 1H), 2.46 (d, *J* = 7.4 Hz, 2H), 2.28 (t, *J* = 7.2 Hz, 2H), 2.12 (ddd, *J* = 15.1, 9.4, 6.8 Hz, 1H), 1.98 (dt, *J* = 14.5, 7.3 Hz, 1H), 1.78 (dtd, *J* = 11.4, 5.8, 2.4 Hz, 1H), 1.61 – 1.47 (m, 2H), 1.45 – 1.32 (m, 2H), 1.29 – 1.15 (m, 14H).

**^13^C NMR** (151 MHz, CDCl_3_) δ 139z.18, 138.38, 137.61, 128.46, 128.15, 126.47, 57.96, 53.11, 41.21, 36.95, 33.29, 30.64, 29.71, 29.12, 28.99, 28.58, 28.43, 28.31, 28.29, 27.91, 27.82, 24.20.

**LRMS** **(APCI)** m/z: [M+H]^+^ calc’d. for C_24_H_37_N_2_O_4_S: 449.6, found 448.8.

1. Cato, M. L., Cornelison, J. L., Spurlin, R. M., Courouble, V. V., Patel, A. B., Flynn, A. R., Johnson, A. M., Okafor, C. D., Frank, F., D'Agostino, E. H., Griffin, P. R., Jui, N. T., and Ortlund, E. A. (2022) Differential Modulation of Nuclear Receptor LRH-1 through Targeting Buried and Surface Regions of the Binding Pocket. *J Med Chem* **65**, 6888-6902

2. Cornelison, J. L., Cato, M. L., Johnson, A. M., D'Agostino, E. H., Melchers, D., Patel, A. B., Mays, S. G., Houtman, R., Ortlund, E. A., and Jui, N. T. (2020) Development of a new class of liver receptor homolog-1 (LRH-1) agonists by photoredox conjugate addition. *Bioorg Med Chem Lett* **30**, 127293
