# Supplementary material for: Comparison of activity, structure, and dynamics of SF-1 and LRH-1 complexed with small molecule modulators
Source: J Biol Chem. 2023 Jun 14;299(8):104921. doi: 10.1016/j.jbc.2023.104921 (PMC10407255; doi:10.1016/j.jbc.2023.104921)
Supplement: Supplementary table 1 [file mmc2.pdf]

**Data collection**	SF-1 + 6N-10CA + TIF2
Space group	$P3_121$
Cell dimensions	
a, b, c (Å)	73.4, 73.4, 194.2
α, β, γ (°)	90, 90, 120
Resolution (Å)	38.59-2.59 (2.68-2.59)
R_{pim}	0.035 (0.453)
I / σI	20.3 (1.21)
$CC_{1/2}$	99.0 (67.0)
Completeness (%)	99.8 (99.2)
Redundancy	12.4 (11.2)
Wilson B-factor	66.5
**Refinement**	
Resolution (Å)	2.59
No. reflections	19623
R_{work} / R_{free}(%)	22.2/28.1
No. atoms	
Protein	3936
Water	13
B-factors	
Protein	82.8
Ligand	70.0
Water	62.7
R.m.s. deviations	
Bond lengths (Å)	0.005
Bond angles (º)	0.68
Ramachandran favored (%)	97.1
Ramachandran outliers (%)	0.21
PDB accession code	8DAF
