# Supplementary figures and images for: Comparison of activity, structure, and dynamics of SF-1 and LRH-1 complexed with small molecule modulators
Source: J Biol Chem. 2023 Jun 14;299(8):104921. doi: 10.1016/j.jbc.2023.104921 (PMC10407255; doi:10.1016/j.jbc.2023.104921)

# Reporter Activity

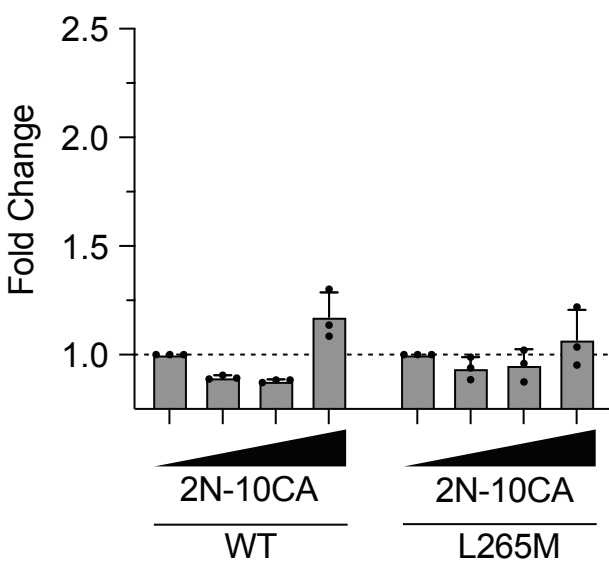

Supplement: Supplementary figure 1 [file mmc3.pdf]

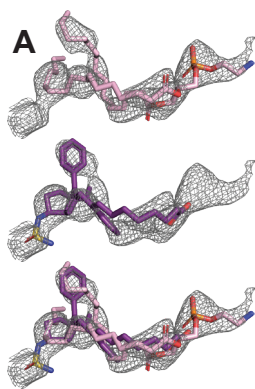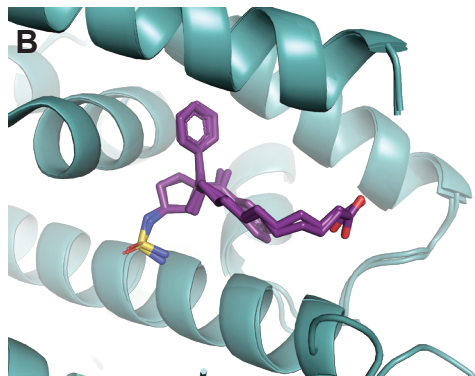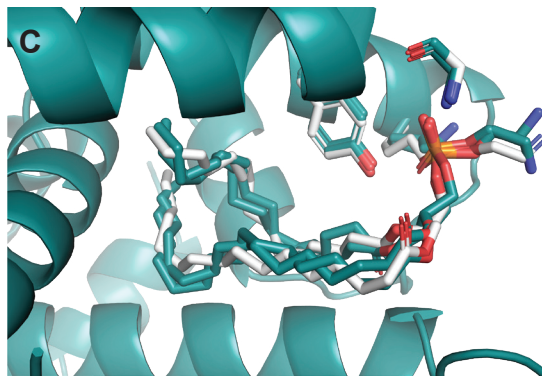

Supplement: Supplementary figure 2 [file mmc4.pdf]

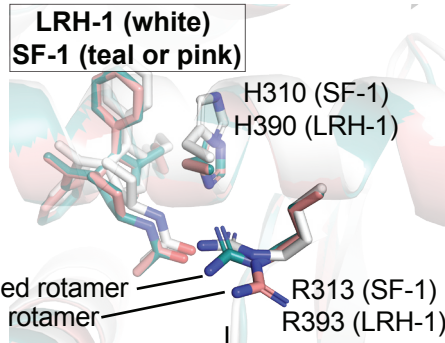

MD Simulation ( $3 \times 500$ -ns)

SF-1-2N (original R313 rotamer)

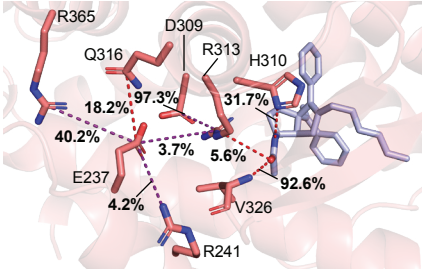

SF-1-2N (repositioned R313 rotamer)

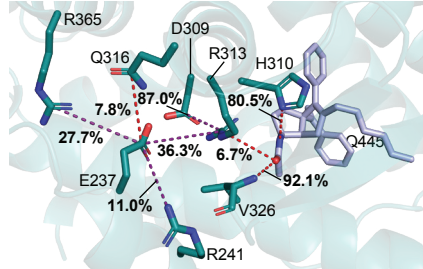

LRH-1-2N

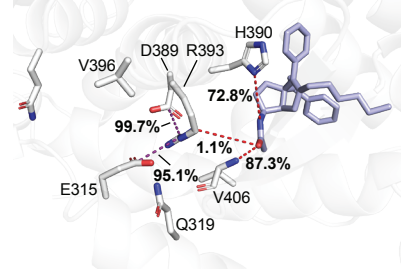

Supplement: Supplementary figure 3 [file mmc5.pdf]
